# Improved myocardial T1 mapping for patients with implanted cardiac devices

**DOI:** 10.1186/1532-429X-18-S1-P25

**Published:** 2016-01-27

**Authors:** Jiaxin Shao, Shams Rashid, Kim-Lien Nguyen, Peng Hu

**Affiliations:** 1 grid.19006.3e0000000096326718Department of Radiological Sciences, David Geffen School of Medicine, University of California, Los Angeles, CA USA; 2 grid.19006.3e0000000096326718Department of Medicine, Division of Cardiology, David Geffen School of Medicine, University of California, Los Angeles, CA USA; 3 grid.19006.3e0000000096326718Biomedical Physics Inter-Departmental Graduate Program, University of California, Los Angeles, CA USA

## Background

Myocardial T1 mapping holds promise for non-invasive evaluation of diffuse fibrosis. Nevertheless, the widely used cardiac T1 mapping techniques, including MOLLI and SASHA, are generally not applicable to patients with implanted cardiac devices, such as implantable cardioverter defibrillators (ICDs) and pacemakers, due to large off-resonance induced by the device and the associated banding artifacts when using bSSFP readouts. We sought to develop and validate an improved technique for accurate myocardial T1 mapping in patients with implanted cardiac devices.

## Methods

**Figure 1:**
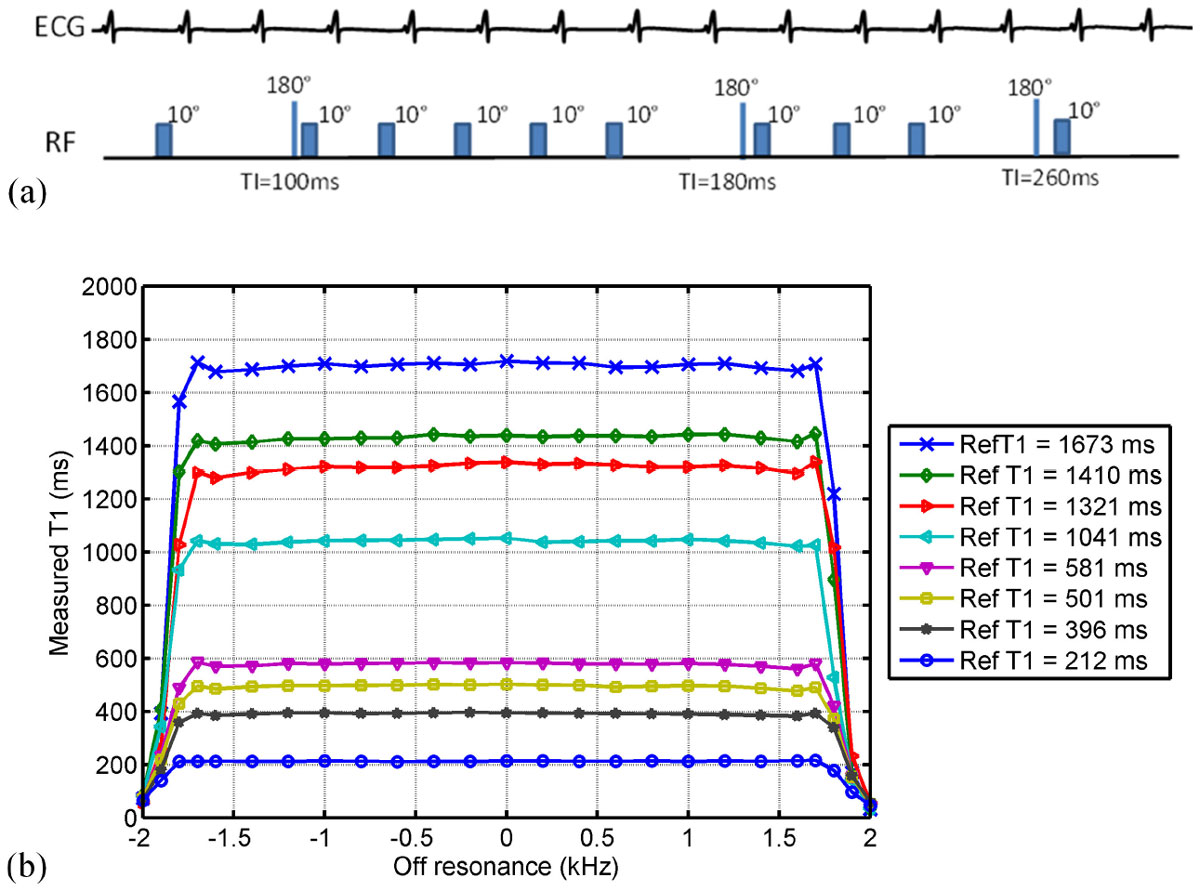
**(a) Illustration of the wideband FLASH-MOLLI acquisition scheme**. The FLASH images were acquired over 13 heart beats during a single breath-hold. (b) Plots of measured T1 using the wideband FLASH-MOLLI as a function of off-resonance in phantom experiments. Wideband FLASH-MOLLI produced consistent T1 estimation over 3.4 KHz. Reference T1 values (Ref T1) were determined using a standard inversion recovery spin-echo sequence.

Recent work by Rashid et al. (Radiology 2014; 270:269-274) demonstrates the use of a wideband adiabatic inversion pulse in late gadolinium enhancement (LGE) MRI to remove hyper-intensity image artifacts during myocardial scar imaging of patients with cardiac devices. In this work, we incorporate a similar wideband inversion pulse to a MOLLI sequence based on FLASH readouts, using a M0-(1)-5-(1)-3-(1)-1 acquisition scheme shown in Figure 1a. Ten FLASH images were acquired over 13 heart beats during a single breath-hold with a resolution of 1.9 × 2.5 × 8.0 mm^3^. The BLESSPC fitting algorithm recently described by Shao et al. (JMRI 2015; doi: 10.1002/jmri.24999) was applied to the reconstruction of T1 maps.

Wideband FLASH-MOLLI was evaluated using 8 phantom studies and validated in 10 healthy volunteers and 5 patients with ICDs using a 1.5T MR scanner. The effects of off-resonance frequency variation, heart rate variation, and presence of ICD on T1 estimation accuracy were investigated using phantom studies. To mimic ICD-induced image artifacts, the ICD generator was attached to the body coil and close to the left shoulder of healthy volunteers. The original FLASH-MOLLI sequence using a 3-(3)-3-(3)-5 acquisition scheme was used to image one healthy volunteer (before and after ICD attachment) and in one patient with ICD for comparison.

## Results

**Figure 2:**
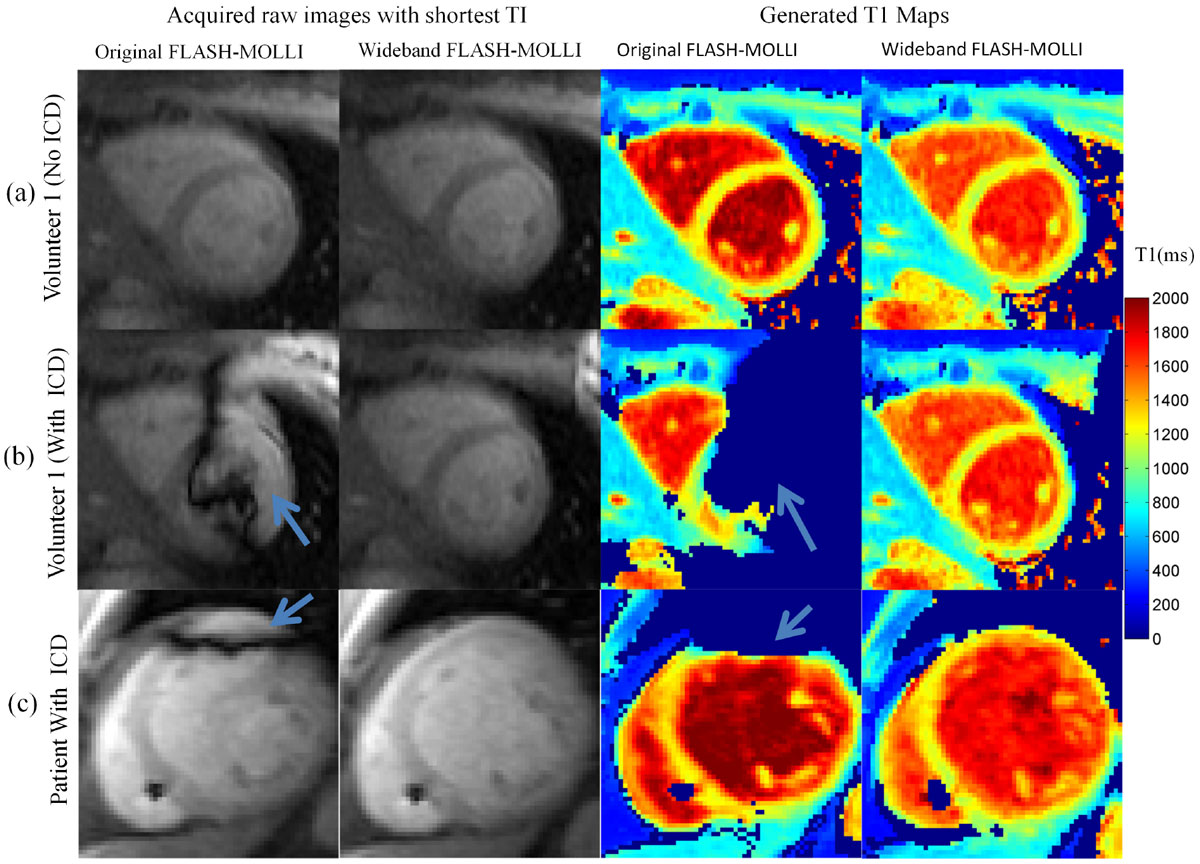
**Example of raw images and corresponding T1 maps acquired in the mid left ventricular short axis plane of a healthy volunteer without (row a) and with (row b) an ICD taped near the left shoulder and of a patient (row c) with an ICD, using the original FLASH-MOLLI (columns 1 and 3) and the wideband FLASH-MOLLI (columns 2 and 4)**. T1 values of pixels where the data did not fit well (R^2^ < 0.95) were set to zero. The raw images acquired using FLASH-MOLLI showed severe dark band artifacts within the myocardium (blue arrow, column 1), which occur at the boundary of inverted and non-inverted regions despite using a FLASH readout. In contrast, the raw images acquired using wideband FLASH-MOLLI (column 2) are without dark band artifacts, resulting in more accurate and homogenous T1 estimation (column 4).

Wideband FLASH-MOLLI generated consistent T1 values over a wide range of off-resonance frequencies (Figure 1b) and showed no dependence on heart rate variation. The maximum T1 estimation errors by wideband FLASH-MOLLI were less than 3% for T1 range from 212 ms - 1673 ms. Compared to original FLASH-MOLLI, wideband FLASH-MOLLI succeeded in 1) removing artifacts resulting from ICD-induced non-uniform signal inversion of the myocardium and 2) producing more homogeneous T1 estimation over the entire myocardium (Figure 2). In all ten healthy volunteers, there was no significant myocardial T1 estimation difference by wideband FLASH-MOLLI before and after ICD attachment (1177 ± 27 ms vs. 1178 ± 27 ms, p = 0.94). Using wideband FLASH-MOLLI, myocardial T1 maps from 5 patients with ICDs were generated without ICD-induced artifacts.

## Conclusions

This study demonstrates the feasibility of using wideband FLASH-MOLLI for accurate myocardial T1 mapping in patients with implanted cardiac devices through mitigation of image artifacts associated with conventional T1 mapping techniques.

